# Crystal Structure and Solid-State Packing of 4-Chloro-5*H*-1,2,3-dithiazol-5-one and 4-Chloro-5*H*-1,2,3-dithiazole-5-thione

**DOI:** 10.3390/molecules26195875

**Published:** 2021-09-28

**Authors:** Christos P. Constantinides, Maria Koyioni, Fadwat Bazzi, Maria Manoli, Daniel B. Lawson, Panayiotis A. Koutentis

**Affiliations:** 1 Department of Natural Sciences, University of Michigan-Dearborn, 4901 Evergreen Rd, Dearborn, MI 48128-1491, USA; bazzihf@umich.edu (F.B.); dblawson@umich.edu (D.B.L.); 2 Department of Chemistry, University of Cyprus, P.O. Box 20537, Nicosia 1678, Cyprus; maria.g.koyioni@gmail.com (M.K.); manoli@minoshis.com (M.M.); koutenti@ucy.ac.cy (P.A.K.)

**Keywords:** 1,2,3-dithiazoles, 1,2,3-dithiazol-5-one, 1,2,3-dithiazole-5-thione, Appel’s salt, thiazoles, dithiazines

## Abstract

The crystal structure and solid-state packing of 4-chloro-5*H*-1,2,3-dithiazol-5-one and two polymorphs of 4-chloro-5*H*-1,2,3-dithiazole-5-thione were analyzed and compared to structural data of similar systems. These five-membered S,N-rich heterocycles are planar with considerable bond localization. All three structures demonstrate tight solid-state packing without voids which is attributed to a rich network of short intermolecular electrostatic contacts. These include S^δ+^…N^δ−^, S^δ+^…O^δ−^, S^δ+^…Cl^δ−^ and S^δ+^…S^δ−^ interactions that are well within the sum of their van der Waals radii (∑_VDW_). B3LYP, BLYP, M06, mPW1PW, PBE and MP2 were employed to calculate their intramolecular geometrical parameters, the Fukui condensed functions to probe their reactivity, the bond order, Bird Index and NICS(1) to establish their aromaticity.

## 1. Introduction

**Scheme 1:**
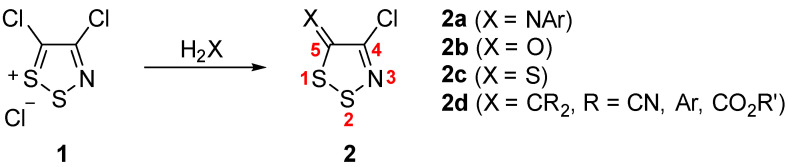
Structure of Appel’s salt **1**; the preparation reaction of neutral 4-chloro-5*H*-1,2,3-dithiazoles **2a–d**; IUPAC numbering of the ring system [3,4,5,6].

4,5-Dichloro-1,2,3-dithiazolium chloride (**1**) (aka “Appel’s salt”), prepared from chloroacetonitrile and disulfur dichloride [1,2], is the main precursor to monocyclic 1,2,3-dithiazoles, and in particular 4-chloro-5*H*-1,2,3-dithiazoles **2** (Scheme 1) [3,4,5,6]. Several 5*H*-1,2,3-dithiazoles display biological activities against bacteria [7,8,9,10], viruses [11,12], fungi/weeds [13,14,15,16,17,18] and cancers [19,20,21]. Furthermore, 4-chloro-5*H*-1,2,3-dithiazoles **2** are useful intermediates in organic synthesis [3,4,5,6]. 1,2,3-Dithiazoles are electrophilic and their chemistry involves reactions of nucleophilic attack at the S1, S2 and C5 atoms [2]. Their chemistry also involves the transformation of their ring system into other systems such as pyrazolo[3,4-*d*]thiazoles [22], pyridothiazoles [23], pyrido[2,3-*d*]pyrimidines [24] and the rare pyrazolo[3,4-*e*][1,2,4]dithiazines and benzo[*e*][1,2,4]dithiazines [25]. 1,2,3-Dithiazoles have also been used as starting materials or appeared as intermediates in various reactions [26,27,28,29,30,31,32,33,34,35,36].

Examining the crystal structures of dithiazoles **2** can enable a better understanding of structure–property relationships. Herein, we report the crystal structures and solid-state packing of 4-chloro-5*H*-1,2,3-dithiazol-5-one (**2b**) and 4-chloro-5*H*-1,2,3-dithiazole-5-thione (**2c**). The latter crystallized as two polymorphs **2c–*α*** and **2c–*β*** with significantly different solid-state packing.

## 2. Results and Discussion

### 2.1. Intramolecular Geometry

Crystals of the dithiazolone **2b** were grown by sublimation under a static vacuum (1.6 Pa) at 30 °C. Dithiazolethione **2c** demonstrated polymorphism: polymorphs **2c–*α*** and **2c–*β*** were obtained by slow evaporation of concentrated solutions in pentane and benzene, respectively. Suitable single crystals of dithiazoles **2b**, **2c–*α*** and **2c–*β*** were then loaded on a goniometer and their crystal structure and solid-state packing were determined at 100 K by single-crystal X-ray diffractometry (Table S1 in Supplementary Information). Below, IUPAC numbering (not a crystallographic one) is used to assist the comparison between the new 5*H*-1,2,3-dithiazoles reported herein and those reported in the literature.

All three dithiazoles are planar. The maximum deviation of the S and N atoms from the plane of the five-membered rings are 0.020 and 0.027 Å, respectively. Their intramolecular geometrical parameters are similar to each other (Table 1) and comparable to those reported for 4-benzoyl-5*H*-1,2,3-dithiazol-5-one (**3**) [37], 4-[*N*-(2-chloroethyl)piperazin-1-yl]-5*H*-1,2,3-dithiazole-5-thione (**4**) [38] and 4-phenyl-5*H*-1,2,3-dithiazole-5-thione polyiodide S–I^+^–S complex (**5**) [39] (Table S2 in Supplementary Information).

The endocyclic S1–S2 bond lengths are 2.0533(7), 2.066(2), 2.058(4)-2.075(4) Å for **2b**, **2c–*α*** and **2c–*β***, respectively (Table 1) and are longer than the S–S bond length 2.034(2) Å reported for Appel’s salt **1** [40]. The short S–S bond in Appel’s salt **1** reflects the significant bond and charge delocalization aiming to achieve a greater aromaticity. Our calculations (Section 2.3.3) indicate that Appel’s salt cation **1** is more delocalized and more aromatic then either the dithiazolone **2b** or dithiazolethione **2c**. The angles at the S atoms range from 93.96(6)° (C5–S1–S2 for **2b**) to 98.23(7)° (S1–S2–N3 for **2b**), all < 120°, are comparable to the analogous angles in previously reported 1,2,3-dithiazol-5-ones/thiones (Table S2 in Supplementary Information).

The C4–N3 bond lengths are 1.269(3), 1.283(6) and 1.23(1)–1.32(1) Å for **2b**, **2c–*α*** and **2c–*β***, respectively (Table 1). These have a pronounced double bond character (1.28 Å) [41] indicating significant localization inside the ring. The S2–N3–C4 internal angles are 115.9(1), 116.2(3) and 114.6(7)-119.6(8)° for 2**b**, **2c–*α*** and **2c–*β***, respectively and are typical of *sp*^2^-hybridized imine N [41]. The S2–N3 bonds lengths are 1.645(2), 1.642(4) and 1.612(9)–1.663(9) Å for **2b**, **2c–*α*** and **2c–*β***, respectively.

The C4–C5 bond lengths are 1.476(2), 1.470(6) and 1.45(2)–1.51(1) Å for **2b**, **2c–*α*** and **2c–*β***, respectively, and are intermediate between typical aromatic C–C bonds (1.41 Å) and C–C single bonds (1.54 Å) [41]. Similar C–C bond lengths have been reported for 1,2,3-dithiazol-5-ones/thiones **3–5** (Table S2 in Supplementary Information).

The C5–O bond length 1.208(2) Å in dithiazolone **2b** is similar to that reported for dithiazolone **3** [37] and is typical of a C=O double bond (1.21 Å) [41]. The angles around C5 (Table 1) support a *sp*^2^-hybridized C of a carbonyl group [41]. The endocyclic C4–C5–S1 angle of 108.7(1)° is narrower and accounts for the five-membered ring strain. The other two C4–C5–O 126.6(2)° and S1–C5–O 124.7(1)° are wider with the one next to the Cl being slightly larger possibly due the steric interactions between the lone pairs of Cl and O atoms.

The C5–X (X=S) bond lengths in **2c–*α*** 1.639(4) Å and **2c–*β*** 1.61(1)–1.65(1) Å are typical C=S double bonds (1.61 Å) [41]. While these bond lengths are similar to the C5–S bond length 1.657(2) Å in dithiazole **4 [38]**, the C5–S bond lengths 1.693(6)–1.694(6) Å in the polyiodide complex of 4-phenyl-5*H*-1,2,3-dithiazole-5-thione (**5**) are slightly longer owing to the delocalization across the S–I^+^–S bridge [39]. The angles around C5 in **2c–*α*** and **2c–*β*** (Table 1) deviate from the expected value of 120° for a *sp*^2^-hybridized C atom but are typical of thiones with one angle narrower and the other two wider, e.g., C4–C5–S1 108.7(3)°, C4–C5–X 126.9(3)° and S1–C5–X 124.3(2)° for polymorph **2c–*α***.

While the bond length of C5=O 1.208(2) Å in the dithiazolone **2b** is significantly different than the bond length of C5=S 1.639(4) Å in thione **2c–*α***, the bond angles around C5 are surprisingly similar (Table 1); C4–C5–S1 108.7(1)°, C4–C5–O 126.6(2)°, S1–C5–O 124.7(1)° for dithiazolone **2b** vs. C4–C5–S1 108.7(3)°, C4–C5–S 126.9(3)° and S1–C5–S 124.3(2)° for thione **2c–*α***.

### 2.2. Crystal Packing and Short Contacts

1,2,3-Dithiazol-5-ones/thiones **2b** and **2c–*α*** crystal pack in the highly symmetrical *Pbca* space group with eight symmetry operators in operation, primarily a series of 2-fold screw axis and glide planes (Table S3 in Supplementary Information). The second polymorph of dithiazolethione **2c–*β*** is of lower symmetry (*P-1*) with only two symmetry operators in effect (identity and inversion).

**Figure 1:**
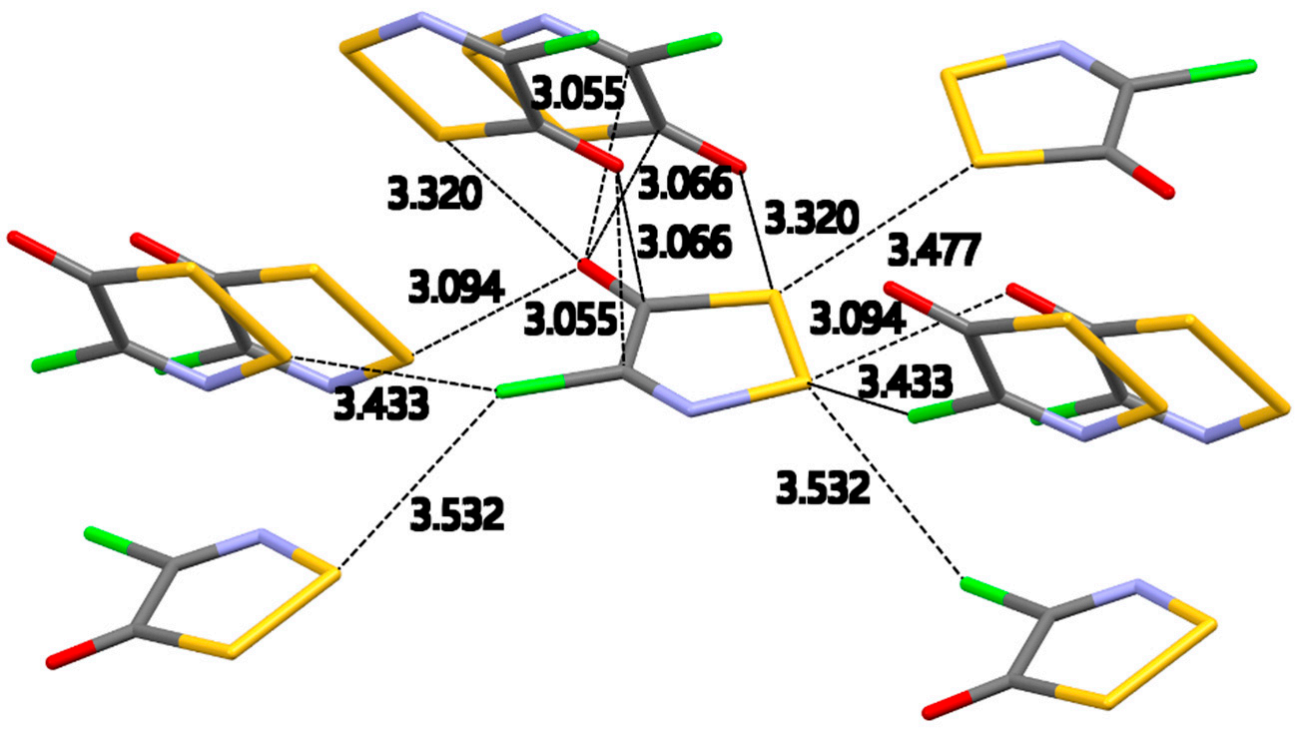
Intermolecular short contacts (Å) expanded for the central dithiazolone **2b** in the asymmetric unit cell.

**Figure 2:**
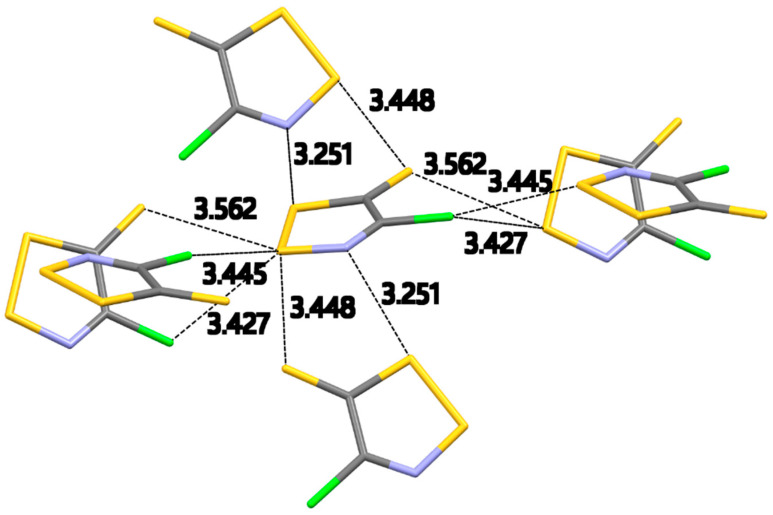
Intermolecular short contacts (Å) expanded for the central dithiazolethione **2c–*α*** in the asymmetric unit cell.

**Figure 3:**
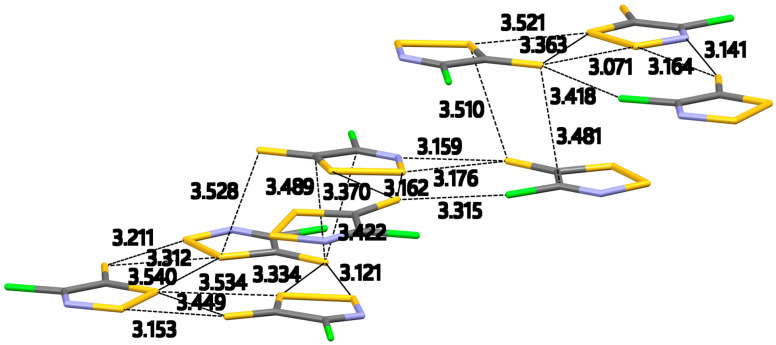
Intermolecular short contacts (Å) for the three supramolecular triangles of dithiazolethione **2c–*β*** formed by the six molecules in the asymmetric unit cell.

There is a rich network of structure-directing intermolecular interactions in the crystal packing of dithiazoles **2b**, **2c–*α*** and **2c–*β*** (Figure 1, Figure 2 and Figure 3). These mainly electrostatic interactions optimize contacts between electronegative and electropositive regions in neighboring molecules. Inside these five-membered rings the S–N and C–N bonds are polar due to the difference in electronegativity of their atoms; 2.58 for S and 3.04 for N and 2.55 for C. The S–N and C–N bonds should therefore be considered polarized in the sense of S^δ+^…N^δ−^ and C^δ+^…N^δ−^. It is expected that the location of two electropositive S atoms next to each other will create a strong electropositive region near the S atoms (Table 2). The presence of lone pairs on the N, Cl and on O and S atoms of the C=O carbonyl and C=S thione groups create pockets of electronegative regions. To better understand the electrostatic contribution to bonding we calculated the molecular electrostatic potential maps (MEP) for dithiazol-5-ones/thiones **2b** and **2c** at the B3LYP/def2-TZVPD level of theory (Table 2); red color corresponds to a maximum negative charge value of −3.0 × 10^−2^ esu, i.e., electronegative character, while blue color corresponds to a maximum positive charge value of 3.0 × 10^−2^ esu, i.e., electropositive character.

The MEP for **2b** and **2c** are as expected blue near the endocyclic electropositive S atoms and red in the vicinity of N, Cl, O, S where the lone pairs of these atoms are located, and a build-up of partial negative charge is expected. Consequently, close intermolecular contacts between the endocyclic S and the rest of the electronegative atoms (N, Cl, O and exocyclic S) should be electrostatically favorable. It should be noted that the exocyclic S atom in **2c** has areas that are red, i.e., negatively charged, where the lone pairs are expected to reside and an area in the center of the atom along the C=S bond axis that is green. Our calculations on Fukui functions (Section 2.3.2) predict an ambivalent chemical behavior which shows the thione S atom to be a site for both nucleophilic and electrophilic chemistry.

Intermolecular interactions are usually considered to be contacts considerably shorter than the sum of the van der Waals radii (∑_VDW_) of the atoms participating in these contacts. They are usually 8–20% shorter than the sum of the equilibrium radii [42]. For some atoms van der Waals radii exhibit significant anisotropy. N and O atoms have almost spherical shapes but for S and Cl the ellipticity and therefore the anisotropy increases [43]. For these anisotropic atoms the minor radii (minor axis ca. 0° or 180°) correspond to contacts close to the plane of the molecule and the major radii (major axis ca. 90°) for contacts perpendicular to the molecular plane [43]. The sum of the minor and major van der Waals radii (∑_VDW_) of the intermolecular contacts present in the crystal packing of **2b**, **2c–*α*** and **2c–*β*** are 3.20, 3.14, 3.18 and 3.20 Å (minor) and 3.63, 3.57, 3.81 and 4.06 Å (major) for S^δ+^…N^δ−^, S^δ+^…O^δ−^, S^δ+^…Cl^δ−^ and S^δ+^…S^δ−^ (endocyclic S to exocyclic thione S), respectively. For the discussion below, we provide both the sum of the minor and major van der Waals radii (∑_VDW_) since the angle of the intermolecular atom approach is somewhere along the 0–180° range.

#### 2.2.1. 4-Chloro-5*H*-1,2,3-dithiazol-5-one (**2b**)

Dithiazolone **2b** crystallized in the *Pbca* space group with one molecule in the asymmetric unit cell. Figure 1 shows all the intermolecular interactions expanded for the central molecule in the asymmetric unit cell. This crystal packing is dominated by S…O and S…Cl intermolecular contacts. The lack of any S…N interactions is surprising but not unexpected as the S…O and S…Cl interactions are potentially stronger and structure directing. The two S…O interactions 3.094(1) Å [∠S–S…O, 89.53(3)°] and 3.320(1) Å [∠S–S…O, 175.00(3)°] are well within the ∑VDW [3.14 Å (minor), 3.57 Å (major)].

There are two S…Cl intermolecular interactions 3.4528(8) Å [∠S–S…Cl, 151.01(3)°] and 3.5316(7) Å [∠S–S…Cl, 130.43(3)°] well within the ∑_VDW_ [3.18 Å (minor), 3.81 Å (major)]. These interactions (Figure 1) are longer than the ionic S…Cl interactions previously reported for Appel’s salt **1** [40].

A triangular and near symmetrical approach of the oxygen atom on top of the C–C bond creates two C…O contacts 3.055(2) Å [∠C–O…C, 119.2(1)°] and 3.066(2) Å [∠C–O…C, 117.7(1)°]. Each carbon atom of the C–C bond has a significant partial positive charge stemming from the polarization of the C–Cl and C=O bonds due to the difference in electronegativity (C^δ+^–Cl^δ−^, C^δ+^=O^δ−^). The location of the oxygen atom is, therefore, ideal to create a bifurcated set of C^δ+^…O^δ−^ contacts (Figure S1a in Supplementary Information). These C…O contacts are within the ∑_VDW_ [3.25 Å (minor), 3.67 Å (major)] [44].

The network of intermolecular contacts for dithiazolone **2b** is concluded with a S…S contact 3.4771(7) Å [∠C–S…S, 149.75(6)°] between two endocyclic S atoms from neighboring molecules. This represents a short contact due to the proximity of the S atoms. Both atoms are expected to have a partial positive charge S^δ+^ but since the electron cloud around S is highly polarizable this could also be an electrostatic interaction.

Molecules of the dithiazolone **2b** stack along the *c*-axis (Figure S1a in Supplementary Information) to form one dimensional (1D) columns. Inside these 1D columns the molecules are connected via S…O 3.320(1) Å and C…O 3.055(2)–3.066(2) Å contacts and arrange in a near herringbone pattern with successive plane angles of 71.36° and 73.46°. Neighboring columns are oriented in a face-to-tail manner and connect via S…O 3.094(1) Å and C…Cl 3.5316 (7) Å contacts to form ladders along the *b*-axis (Figure S1a in Supplementary Information). Two parallel chains with the same direction (Figure S1b in Supplementary Information) form a ribbon along the *bc-*diagonal. An antiparallel ribbon completes the 2D sheet along the *bc*-diagonal.

#### 2.2.2. 4-Chloro-5*H*-1,2,3-dithiazole-5-thione (**2c–*α***)

The *α*-phase of dithiazolethione **2c–*α*** crystallized in the *Pbca* space group with one molecule in the asymmetric unit cell. Figure 2 shows all the intermolecular interactions expanded for the central molecule in the asymmetric unit cell. This crystal packing is dominated by S…Cl and S…S intermolecular contacts. In the absence of strong structure directing S…O contacts, S…N interactions appear in the crystal packing. There is only one S…N interaction 3.251(4) Å [∠S–S…N, 166.29(9)°] well within the ∑_VDW_ [3.20 Å (minor), 3.63 Å (major)].

The crystal packing of S,N-rich heterocycles is dominated by the presence of short S…N intermolecular contacts. S-N bonds are strongly polar (S^δ+^…N^δ−^) and therefore electrostatically favored.

The planar geometry of this molecule results in a short intramolecular S…N contact of 2.597(2) Å, well below the minor ∑_VDW_ (3.20 Å). Neighboring molecules form centrosymmetric pairs linked by short S…N interactions of 3.035(2) Å. No S…N contacts appear in the reported crystal structures of the benzoyldithiazolone **3** and dithiazolethiones **4** and **5** [37,38,39].

There are two S…Cl intermolecular interactions of 3.427(1) Å [∠S–S…Cl, 109.88(5)°] and 3.445(1) Å [∠S–S…Cl, 140.66(6)°] well within the ∑VDW [3.18 Å (minor), 3.81 Å (major)]. This is a near triangular interaction of the same covalently bound Cl to two S atoms of neighboring dithiazoles (Figure 2). The distances of the S…Cl contacts in **2c–*α*** are similar to those in dithiazolone **2b**. It should be noted that while dithiazolethione **4** has a terminal C–Cl bond, no S…Cl contacts appear in its crystal packing [38]. Instead, there is a highly symmetrical bifurcated S…S contact of 3.3894(7) and 3.3143(8) Å between the exocyclic C=S sulfur atom and the endocyclic S–S atoms [38]. The thione C=S bond is weakly polar and easily polarizable as sulfur is slightly more electronegative than carbon (2.58 for S vs. 2.55 for C) and is therefore expected to bear partial negative charge. The S^δ+^…S^δ−^ contacts between the endocyclic S–S atoms and the exocyclic thione S atom in compound **4** are partially of electrostatic nature. These S…S contacts are also present in the crystal structure of **2c–*α*** albeit with a different geometry. The bifurcation of the thione S atom extends on S atoms of S–S bonds in neighboring rings (Figure 2). These S…S contacts of 3.562(2) Å [∠N–S…S, 159.8(2)°] and 3.448(2) Å [∠S–S…S, 153.43(6)°] are similar to the ones previously reported in compound **4**. The S…S contact for **2c–*α*** is 3.328(1) Å [∠C–S…S, 85.8(1)°] between two endocyclic S atoms from neighboring molecules. A similar interaction appears in the crystal packing of dithiazolone **2b**.

Molecules of the dithiazolethione **2c–*α*** form zig-zag chains along the *a*-axis (Figure S2a in Supplementary Information). Inside these chains successive molecules are arranged sideways with opposite ends and are connected via two short S…N and S…S contacts of 3.251(4) and 3.448(2) Å, respectively. The zig-zag chains pack parallel along the *b*-axis (Figure S2b in Supplementary Information) and are connected via S…S contacts of 3.328(1) Å to form a tight brick wall of dithiazolethiones without any voids. The packing along the *c*-axis (Figure S2c in Supplementary Information) resembles those in the crystal packing of dithiazolone **2b**. Chains along the *c*-axis are formed by molecules that are organized in a head-to-tail orientation and are connected by S…S and S…Cl contacts of 3.562(2) and 3.427(1) Å, respectively. Two chains, linked by S…Cl contact of 3.445(1) Å, run parallel across *c*-axis to form a ribbon. Neighboring ribbons run antiparallel to each other and are connected by S…S and S…N contacts of 3.448(2) and 3.251(4) Å, respectively, to form a 2D sheet (Figure S2c in Supplementary Information).

#### 2.2.3. 4-Chloro-5*H*-1,2,3-dithiazole-5-thione (**2c–*β***)

The *β*-phase of dithiazolethione **2c–*β*** crystallized in the *P-1* space group with six molecules in the asymmetric unit cell and three crystallographically independent trimers. Figure 3 shows the formation of supramolecular triangles and all the intermolecular interactions expanded for the molecules in the asymmetric unit cell. This crystal packing is dominated by S…Cl, S…N and S…S contacts. Inside the supramolecular triangles, molecules of **2c–*β*** are arranged around a non-crystallographic three-fold axis and are connected by a series of in-plane contacts (Figure 3). The supramolecular triangles pack next to each other to form a planar infinite 2D sheet (Figure S3a in Supplementary Information). The contacts inside and between the triangles are not of equal length due to the low symmetry of the crystal. While each triangle has the same number and type of contacts, their length varies.

The thione S atoms in **2c–*β*** form bifurcated contacts with the S–S atoms of the dithiazole ring (Figure 3). These S…S contacts are in the range of 3.071(4)–3.370(4) Å [∠C–S…S, 96.7(4)–136.6(4)°] and are well within the ∑_VDW_ [3.20 Å (minor), 4.06 Å (major)]. While this type of interaction is also seen in **2c–*a***, the highly polarizable nature of sulfur allows for the formation of interactions between the exocyclic thione S atom and the more electronegative N and Cl atoms. This type of interaction originates from the positive *σ* hole of the S atom. In the C=S bond, some of the electronic charge of the S atom is polarized toward the bond region, leading to a redistribution of electronic density from its outer region (along the extension of the bond) on its equatorial sides [45]. Therefore, negative electrostatic potential is developed around the sites of the S atom while its outer portion along the C=S bond becomes more positive (*σ* hole). This is evident from the MEP of dithiazolethione **2c** (Table 2) where red areas of negative charge density are located on the sides of the S atom and a green area in the outer region of the bond axis. This *σ* hole on the thione S atom is potentially responsible for the formation of the S…N and S…Cl interactions. The S…N interactions in **2c–*β*** are formed by the exocyclic thione S atom instead of the endocyclic S–S atoms like in **2c–*a***. These S…N interactions are in the range of 3.14(1)–3.31(1) Å [∠C–S…N, 163.4(4)–157.8(4)°] and well within the ∑VDW [3.20 Å (minor), 3.63 Å (major)]. The exocyclic thione S atom also formed an interaction with a Cl atom. This S…Cl contact is in the range of 3.315(4)–3.418(4) Å [∠C–S…Cl, 150.3(4–153.4(4)°]. The set of close intermolecular interactions in **2c–*β*** is completed with a Cl…Cl contact of 3.337(4)–3.342(4) Å [∠C–Cl…Cl, 164.3(4)–164.5(4)°] not present in the crystal packing of **2c–*a*** or compounds **2b**, **3–5**. This interaction might be an outcome of the proximity of the dithiazolethiones.

The 2D sheets formed by the supramolecular triangles, pack parallel to the *ac*-diagonal and while well separate from each other (Figure S3b in Supplementary Information) they are connected via short S…S and S…C contacts of 3.479(4)–3.528(4) Å and 3.42(1)–3.49 (1) Å, respectively.

### 2.3. DFT Calculations

#### 2.3.1. Computational Bond Length Analysis

Table 3 provides experimental, B3LYP, BLYP, M06, mPW1PW, PBE and MP2 calculated bond lengths for the dithiazolone **2b**. Referring to the bond length data, both DFT and MP2 bond lengths compare well with the experimental values. For example, the B3LYP and BLYP C5–S1 bond length has the greatest difference from experimental value where B3LYP is short by 0.038 Å or 2.1% and BLYP is long by 0.079 Å or 4.5% (Table S4 in Supplementary Information). M06 shows a mild improvement as the C5–S1 bond length is longer by 0.036 Å than the experimental one or overall 2.0%. The results are similar for mPW1PW, PBE and MP2. The S1–S2, N3–C4 and C5–O bond lengths are accurately predicted by PBE, M06 and BLYP, respectively, as these methods provide calculated bond lengths within the experimental range.

In addition to a basic comparison of experimental bond lengths and bond angles to the measured X-ray crystallography values we also provide a comparison by estimated standard deviation. In this approach we determine whether two bonds are significantly different. The convention is that measured values are said to be ‘significantly different’ if the difference in their lengths is greater than three times the weighted standard deviation (WSD).

Table S5 in Supplementary Information provides an assessment of the difference between the experiment and computational approach performance in terms of a multiple of the weighted standard deviation (WSD). A value > 3 or <−3 indicates a significant difference between the computed bond length and the experimental one. Table S5 shows that the C5–S1 is consistently the most challenging for all the computational methods as these values range from 39.5 times the WSD using BLYP to 9.5 times the WSD for PBE and MP2. The calculated S1–S2 bond has a large range where the worst case is 75.3 times the WSD using BLYP but only −0.4 times the weighted standard deviation for PBE.

Table 4 provides a comparison between the experimental bond angles and the DFT and MP2 bond angles of the dithiazolone **2b**. The difference between the experimental bond angles and computed bond angles are mostly < 1° except for the S2–N3–C4 bond angle. This latter bond angle ranges from −1.6% error for B3LYP and M06 to −0.2% for MP2. On average the differences of either the DFT or MP2 are < 1% (Table S6 in Supplementary Information).

Table S7 in Supplementary Information provides the same assessment of the bond angles as was provided in Table S5 for the bond lengths.

As with the bond lengths, a value > 3 or <−3 indicates a significant difference between the computed bond angle and the experimental value. The selected computational methods perform well for many of the bond angles except the S1–C5–O where the multiple of the WSD ranges from −15.0 for BLYP to a value of −7.9 for MP2. The C5–S1–S2 bond angle also has a large range of WSD from −19.2 for BLYP to 3.3 for MP2. The biggest discrepancy for all methods is observed for the S2–N3–C4 bond angle, whereas the calculated C4–C5–S1 bond angles are all within 3 times the WSD for every computational method used.

Table 5 provides experimental and computed bond lengths of the dithiazolethione **2c** and Table S8 in Supplementary Information provides the percent differences. Replacement of the O in the dithiazolone **2b** by S in thione **2c** does little to the relative differences between our crystallographic bond lengths and the computed bond lengths. Once again B3LYP and BLYP obtain a C5–S1 bond length only slightly longer than the experimental value, by 0.009 and 0.032 Å, respectively. The largest relative difference for B3LYP and BLYP is the S1–S2 bond where B3LYP is 0.029 Å shorter or 1.4% and BLYP is 0.064 Å shorter or 3.1%. This is not true for the other functionals or for MP2. Rather the largest difference between experiment and calculated mPW1PW and PBE is in the S2–N3 bond length but these are all only 1% shorter. MP2 provides the most significant deviation from the experimental bond lengths in the N3–C4 bond where the computational difference is longer by 0.026 Å or 2.0%. Arguably, there are more calculated bond lengths within the experimental range for **2c** rather than **2b**.

In terms of the WSD, the S1–S2 is well above 3 for B3LYP, BLYP and M06, below −3 for MP2 and is 0.1 for mPW1PW and −1.2 for PBE (Table S9 in Supplementary Information). Arguably, mPW1PW and PBE do quite well at determining the bond lengths where only the WSD of S2–N3 is below −3 or −4.3 for mPW1PW and −4.4 for PBE and the WSD of C4–Cl is −3.7 for mPW1PW and −4.1 for the PBE.

Table 6 gives the experimental and the DFT and MP2 calculated bond angles of the dithiazolethione **2c**. The difference between experimental bond angles and computed bond angles are, with a few exceptions, nearly 1° of experiment (Table S10 in Supplementary Information). The notable exception is the S1–C5–S angle where the difference between experiment and calculation is 2.1°, 2.8°, 2.1°, 1.7°, 1.7° and 1.6° smaller for the B3LYP, BLYP, M06, mPW1PW, PBE and MP2, respectively. On average the percentage difference between the experimental and computational bond angles for each method are <0.1° (Table S10).

Table S11 in Supplementary Information provides the WSD of the computed bond angles for of the dithiazolethione **2c**. MP2 performs closest to 3 × WSD of angle S1–C5–S being −7.9 up to 4.1 for S1–S2–N3. Of the DFT methods, PBE compares favorably with MP2 in that this DFT method produces angles with WSD that range from −8.6 for S1–C5–S to 5.1 for the N3–C4–Cl.

While crystal packing can influence the ring geometries, our gas phase calculations reproduce the experimental bond lengths and angles within a few percent. Thus, the gas phase computations provide a useful analysis of the electronic properties of the rings.

#### 2.3.2. Fukui Condensed Functions

We calculated the condensed electrophilic (*f* ^+^), nucleophilic (*f* ^−^) and radicalary attack (*f* ^0^) Fukui functions based on NBO population analysis [46] with the B3LYP, BLYP, M06, mPW1PW and PBE1PBE functionals for both dithiazolone **2b** (Tables S12–S14 in Supplementary Information) and the dithiazolethione **2c** (Tables S15–S17 in Supplementary Information). Fukui functions act as reactivity indices that give information about which atoms in a molecule can either loose or accept an electron [47]. The information allows the determination of atoms more prone to undergo nucleophilic or electrophilic attack, e.g., the atom with the largest *f* ^+^ value indicates where the onset of a nucleophilic reaction will take place. The calculated Fukui functions of each atom in the ring are consistent and of similar value among the various methods we used (Tables S12–S17). Each condensed Fukui function clearly identified a preferred site of nucleophilic or electrophilic attack.

**Figure 4:**
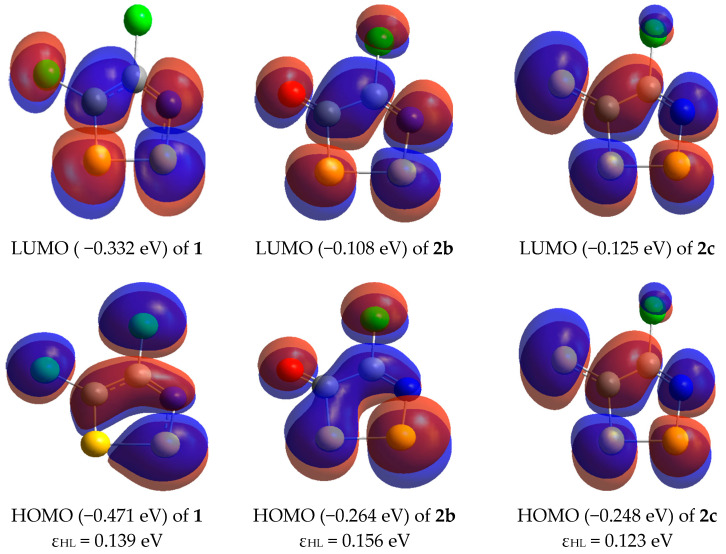
HOMO and LUMO orbitals calculated at the B3LYP/def2-TZVPD level of theory along with their energies (eV) and the ε_HL_: HOMO-LUMO gap (eV) for Appel’s salt **1**, dithiazolone **2b** and dithiazolethione **2c**.

For the dithiazolone **2b**, the site of highest electrophilicity is the S2 atom (*f* ^+^ 0.305–0.353, Table S12) followed by the S1 atom (*f* ^+^ 0.209–0.223). This is not surprising as sulfur is easily polarizable and S2 is involved in the weakest bonds (S–S and S–N; 430.03 and 467 kJꞏmol^−1^, respectively [48]) of the heterocycle. Interestingly, the S2 atom is also most prone to radicalary attack (*f* ^0^ 0.249–0.268, Table S14). The LUMO of dithiazolone **2b** has significant orbital density on both S2 and S1 atoms (Figure 4). These two S atoms show considerable blue coloration (positive electrostatic potential) in the MEP (Table 2) further supporting their electrophilic nature. Reactions of dithiazolone **2b** with nucleophiles are expected to proceed via attack at either S2 or S1 as these atoms have similar *f* ^+^ values. These reactions will proceed with probable fragmentation of the S–S or S–N bonds, i.e., cleavage of the heterocycle. The high electrophilic Fukui functions (*f* ^+^) on S2 and S1 support the observed intermolecular interactions. As discussed above (Section 2.2.1), the crystal packing of **2b** is dominated by S…O and S…Cl intermolecular contacts. A closer look at Figure 1 and Figure S1 in SI show that both S atoms act as acceptors of electron density from the lone pairs of O and Cl. In particular, the S2 atom is participating in both S…O and S…Cl contacts while S1 only in S…O. In reactions with electrophiles, dithiazolone **2b** will have the possibility to react from either S atoms (*f* ^−^ 0.181–0.192 for S1 and *f* ^−^ 0.182–0.193 for S2) or from N3 (*f* ^−^ 0.160–0.172) since these three atoms have near equal nucleophilicities.

In contrast to dithiazolone **2b**, the dithiazolethione **2c** appears to be most prone to either nucleophilic, electrophilic and radical attack at the exocyclic thione S atom with *f* ^+^, *f* ^−^ and *f* ^0^ of ~0.3–0.4 (Tables S15–S17). In support, both the HOMO and LUMO of **2c** have significant orbital density on the thione S atom (Figure 4). The MEP (Table 2) shows both red and green coloration for the thione moiety, a consequence of a potential *σ* hole formation, indicating the ambivalent behavior of this exocyclic sulfur atom [49]. This is further evident in the intermolecular contacts of polymorphs **2c–*α*** and **2c–*β***. In **2c–*α*** the exocyclic thione S atom behaves as a nucleophile donating its lone pairs to form contacts with the endocyclic electron poor S–S atoms. While this type of interaction is also present in the crystal packing of **2c–*β***, the ambivalent character of the thione S atom is evident by the formation of S…Cl and S…N contacts wherein the thione S atom behaves as an electrophile through its *σ* hole, accepting contacts from the lone pairs of Cl and N.

We also calculated the condensed Fukui functions for Appel’s salt **1** (Tables S18–S20 in Supplementary Information) and found that S2 is the site of highest electrophilicity. This agrees with the prominent positive charge for S2 recently reported by Bartashevich and coworkers [50]. Our Fukui calculations indicate that S1 is the site of highest activity towards radical attack and the two Cl atoms are the sides of high nucleophilicity.

#### 2.3.3. Bond Order, Bird Index and NICS(1)

The Wiberg bond orders [51] are calculated within the NBO analysis. This approach to computing bond orders results in values usually close to formal bond order for most chemical systems. Table 7 provides the B3LYP/def2-TZVPD bond orders for the Appel’s salt **1**, dithiazolone **2b** and the dithiazolethione **2c**. The bond orders of the Appel’s salt **1** reflect the delocalization around the ring where the S1–S2 has a bond order of 1.08 and is the least delocalized of all of the bonds. The bond orders of the dithiazolone **2b** and the dithiazolethione **2c** show significantly less delocalization. In dithiazolone **2b** the N3–C4 bond order is 1.68 and in dithiazolethione **2c** the analogous bond order is 1.64. In dithiazolone **2b** the C5–O bond order is 1.77 and in dithiazolethione **2c** the analogous bond order albeit the C5–S is 1.66. These bond orders suggest significant double bond character. By contrast, the analogous bonds in the Appel’s salt **1** have bond orders of 1.48 for the N3–C4 and 1.22 for the C5–Cl2, i.e., less double bond character. The S2–N3 of the dithiazolone **2b** has a bond order of 1.12 and the same bond order in the dithiazolethione **2c** is 1.13 suggesting a single bond. The same is true for the C5–S1 bond orders in both the dithiazolone **2b** and dithiazolethione **2c** where the values are 1.06 and 1.15, respectively. The latter bond orders are larger in the Appel’s salt **1** being 1.29 and 1.36, respectively. According to the bond orders, Appel’s salt **1** is more delocalized than either the dithiazolone **2b** or dithiazolethione **2c**. The experimental and calculated bond lengths and bond angles of Appel’s salt **1** can be found in Tables S21 and S22 (Supplementary Information). Our calculated NBO bond orders for Appel’s salt **1** compared favorably with the recently reported QTAIM-based bond orders [50]. The latter were calculated using experimental electron density. The NBO and QTAIM-based bond orders for the endocyclic bonds C5-S1 (1.36 vs. 1.364), S1-S2 (1.08 vs. 0.912), S2-N3 (1.29 vs. 1.325) are in a good agreement but less so for N3-C4 (1.48 vs. 1.234) and C5-C4 (1.21 vs. 0.917).

To further explore the delocalization, we computed the Bird Index (*I*_n_) [52] along with the nucleus-independent chemical shifts (NICS) [53,54]. The Bird Index, *I*_n_ evaluates the aromaticity of a ring in terms of the statistical evaluation of the deviations in peripheral bond orders. NICS(1) evaluates the aromaticity of cyclic systems and is defined as the negative value of the absolute shielding computed at 1 Å above the ring centroid. From the experimental bond lengths our computed *I*_n_ for dithiazolone **2b** is 36.1, for the dithiazolethione **2c** is 39.9 and for the Appel’s salt **1** is 62.2 (*I*_5_ = 38 for oxazole and *I*_5_ = 62 for 1,2-dithiolium [52]). Using the B3LYP bond orders as shown in Table 7, the resulting Bird Indices are only slightly larger or 37.5 for dithiazolone **2b** 44.3 for dithiazolethione **2c** and 63.6 for Appel’s salt **1**.

Table 8 provides the NICS(1) for the Appel’s salt **1**, dithiazolone **2b** and dithiazolethione **2c**. Comparison of the Appel’s salt **1** with dithiazolone **2b** and dithiazolethione **2c** suggests the Appel’s salt cation to be more aromatic than either dithiazolone **2b** or dithiazolethione **2c**. Using the NICS(1) as an index of the aromaticity, the relative ordering of the compounds in terms of aromaticity of the *π* electrons is Appel’s salt **1** > dithiazolone **2b** > dithiazolethione **2c**.

## 3. Conclusions

We examined the intramolecular crystal structure and solid-state packing of two neutral monocyclic 4-chloro-5*H*-1,2,3-dithiazoles, the 5-one **2b** and the two polymorphs of 5-thione **2c** (**2c–*α*** and **2c–*β***). Both molecules are planar and the intramolecular geometrical parameters support localization of bonds inside the ring. Their crystal packing is dominated by S…N, S…O, S…Cl and S…S short intermolecular interactions of primarily electrostatic nature. Condensed Fukui functions indicate electrophilic sites at S2 and at a lesser degree on S1 for the dithiazolone **2b** and the thione exocyclic S for the dithiazolethione **2c**. The nucleophilic sites for dithiazolone **2b** are S1, S2 and N3 on par and the exocyclic S thione for **2c**. The ambivalent character of the S thione in **2c** is evident by the rich intermolecular contacts it participates with both positive (endocyclic S–S atoms) in polymorph **2c–*α*** and negative (O, N, Cl) atoms in **2c–*β***. This can be best explained by the formation of a *σ* hole which polarizes the S atom in regions of negative and positive electron density. Bond orders, Bird Indices and NICS(1) calculations showed that dithiazolone **2b** and dithiazolethione **2c** do not have an extensive delocalization and are, therefore, less aromatic that Appel’s salt **1**.

## 4. Experimental

### 4.1. Single-Crystal X-ray Diffraction

Data were collected on an Oxford-Diffraction Supernova diffractometer, equipped with a CCD area detector utilizing Cu-Kα radiation (*λ* = 1.5418 Å) for **2b** and **2c–*α*** and Mo-Kα radiation (*λ* = 0.71073 Å) for **2c–*β***. Suitable crystals were attached to glass fibers using paratone-N oil and transferred to a goniostat where they were cooled for data collection. Unit cell dimensions were determined and refined by using 946 (9.19 ≤ *θ* ≤ 71.90°) reflections for **2b**, 944 (6.65 ≤ *θ* ≤ 66.96°) reflections for **2c–*α*** and 3131 (3.25 ≤ *θ* ≤ 27.36°) reflections for **2c–*β***. Empirical absorption corrections (multi-scan based on symmetry-related measurements) were applied using CrysAlis RED software [55]. The structures were solved by direct method and refined on *F*^2^ using full-matrix least squares using SHELXL97 [56]. Software packages used: CrysAlis CCD [55] for data collection, CrysAlis RED [55] for cell refinement and data reduction, WINGX for geometric calculations [57], and DIAMOND [58] for molecular graphics. The non-H atoms were treated anisotropically.

#### 4.1.1. 4-Chloro-5*H*-1,2,3-dithiazol-5-one (**2b**)

Crystal refinement data (CCDC 2106260): C_2_ClNS_2_O, *M* = 153.60, Orthorhombic, space group *P b c a*, *a* = 5.6128(3) Å, *b* = 12.4796(9) Å, *c* = 14.3419(9) Å, *α* = *β = γ* = 90°, *V* = 1004.59(11) Å^3^, *Z* = 8, T = 100(2) K, *ρ*_calcd_ = 2.031 gꞏcm^−3^, 2*θ*_max_ = 72. Refinement of 64 parameters on 982 independent reflections out of 3093 measured reflections (*R*_int_ = 0.0246) led to *R*_1_ = 0.0261 [I > 2*σ*(I)], *wR*_2_ = 0.0663 (all data), and *S* = 1.112 with the largest difference peak and hole of 0.264 and −0.381 e^−3^, respectively.

#### 4.1.2. 4-Chloro-5*H*-1,2,3-dithiazole-5-thione (**2c–α**)

Crystal refinement data (CCDC 2106261): C_2_ClNS_3_, *M* = 169.66, Orthorhombic, space group *P b c a*, *a* = 11.0262(5) Å, *b* = 7.2698(3) Å, *c* = 13.3111(8) Å, *α* = *β = γ* = 90°, *V* = 1067.00(9)Å^3^, *Z* = 8, T = 100(2) K, *ρ*_calcd_ = 2.112 g cm^−3^, 2*θ*_max_ = 67. Refinement of 64 parameters on 953 independent reflections out of 5321 measured reflections (*R*_int_ = 0.1001) led to *R*_1_ = 0.0680 [I > 2*σ*(I)], *wR*_2_ = 0.1993 (all data), and *S* = 1.077 with the largest difference peak and hole of 0.836 and −1.097 e^−3^, respectively.

#### 4.1.3. 4-Chloro-5*H*-1,2,3-dithiazole-5-thione (**2c–β**)

Crystal refinement data (CCDC 2106262): C_2_ClNS_3_, *M* = 169.66, Triclinic, space group *P-1*, *a* = 7.113(5) Å, *b* = 13.323(5) Å, *c* = 18.343(5) Å, *α* = 76.886(5)°, *β =* 82.914(5)°, *γ* = 76.429(5)°, *V* = 1641.2(14) Å^3^, *Z* = 12, T = 100(2) K, *ρ*_calcd_ = 2.060 g cm^−3^, 2*θ*_max_ = 25. Refinement of 379 parameters on 5782 independent reflections out of 10938 measured reflections (*R*_int_ = 0.0911) led to *R*_1_ = 0.0845 [I > 2*σ*(I)], *wR*_2_ = 0.2120 (all data), and *S* = 0.981 with the largest difference peak and hole of 0.228 and −0.806 e^−3^, respectively.

### 4.2. Computational Methodology

Our crystallographic data provided the initial geometry for the pair of structures. Each structure was energy optimized by density functional theory. The combinations of exchange and correlation functionals used in this work include B3LYP [58,59,60,61,62], BLYP [59,63], mPW1PW [64], PBE1PBE [65] and M06 [66] as implemented in Gaussian09 [67]. The methods used in this work are proven to represent similar heterocyclic compounds. B3LYP is a standard of DFT for gas phase molecules and is a well proven compromise between computational cost and accuracy [68,69]. BLYP and PBE are also common functionals applied to gas phase molecules and yield comparable results where the difference is that BLYP and PBE are gradient corrected functionals and B3LYP is a hybrid function. These approaches have been shown to provide good results for magnetic, vibrational, and electronic properties of molecules as compared to DFT functionals that include extensive parameterization [70]. The mPW1PW fits the exact exchange energy of isolated atoms and the differential exchange of ideal gas dimers significantly improving the long-range behavior of the exchange functional [71]. The M06 functional introduces empirically optimized parameters into the exchange-correlation functional and provides good geometry, energy and property data [66]. MP2 is a standard ab initio computational method that includes correlation and is free from the spurious self-interaction of electrons and naturally includes dispersion. The basis set utilized in this work consisted of a single def2-TZVPD. This basis is optimized for properties and computational cost specifically for density functional theory [72]. The calculated nucleophilic (*f* ^−^), electrophilic (*f* ^+^) and radical attack (*f* ^0^) characterize the electron reorganization that results in site electrophilic or nucleophilic activation/deactivation [47]. Calculation of the Fukui reactivity-indices was performed for each DFT from the NBO [46] populations using the UCA-FUKUI software [73].

## Figures and Tables

**Table 1:**
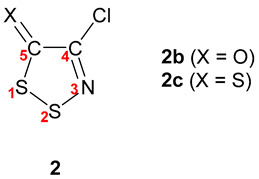
Intramolecular geometrical parameters for dithiazolone **2b** and thiones **2c–*α*** and **2c–*β***.

Compound.	Bonds	Bond Length (Å)	Angles	Bond Angles (°)
**2b** (X = O)	C5–S1	1.774(2)	C5–S1–S2	93.96(6)
	S1–S2	2.0533(7)	S1–S2–N3	98.23(7)
	S2–N3	1.645(2)	S2–N3–C4	115.9(1)
	N3–C4	1.269(3)	N3–C4–C5	123.1(2)
	C5–C4	1.476(2)	N3–C4–Cl	120.5(1)
	C5–X	1.208(2)	C5–C4–Cl	116.3(1)
	C4–Cl	1.715(2)	C4–C5–S1	108.7(1)
			C4–C5–X	126.6(2)
			S1–C5–X	124.7(1)
**2c–*α*** (X = S)	C5–S1	1.740(4)	C5–S1–S2	95.2(1)
	S1–S2	2.066(2)	S1–S2–N3	96.8(2)
	S2–N3	1.642(4)	S2–N3–C4	116.2(3)
	N3–C4	1.283(6)	N3–C4–C5	123.0(4)
	C5–C4	1.470(6)	N3–C4–Cl	118.0(3)
	C5–X	1.639(4)	C5–C4–Cl	119.0(3)
	C4–Cl	1.718(4)	C4–C5–S1	108.7(3)
			C4–C5–X	126.9(3)
			S1–C5–X	124.3(2)
**2c–*β*** (X = S) *^a^*	C5–S1	1.70(1)–1.75(1)	C5–S1–S2	94.7(4)–95.9(4)
	S1–S2	2.058(4)–2.075(4)	S1–S2–N3	95.6(3)–97.4(3)
	S2–N3	1.612(9)–1.663(9)	S2–N3–C4	114.6(7)–119.6(8)
	N3–C4	1.23(1)–1.32(1)	N3–C4–C5	119.2(9)–124(1)
	C5–C4	1.45(2)–1.51(1)	N3–C4–Cl	117.0(8)–122.4(8)
	C5–X	1.61(1)–1.65(1)	C5–C4–Cl	116.1(8)–119.8(8)
	C4–Cl	1.69(1)–1.73(1)	C4–C5–S1	107.6(7)–110.4(8)
			C4–C5–X	125.0(8)–128.3(8)
			S1–C5–X	122.4(6)–125.3(7)

*^a^* Bond lengths and angles are the range of values found in six molecules of the asymmetric unit cell.

**Table 2:** B3LYP/def2-TZVPD potential energy maps (MEP) of the dithiazolone **2b** and of the thione **2c**; the isovalue for both surfaces is 0.001.

Perspective	2b	2c
Above the plane of the ring (translucent)	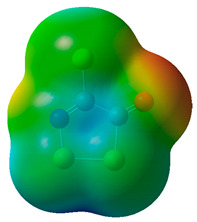	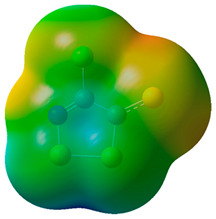
Above the plane of the ring	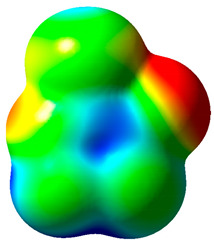	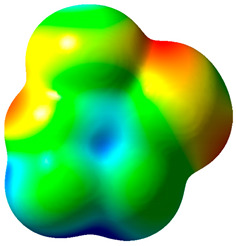
In plane, across the N–Cl–X atom axis (X= O and S for **2b** and **2c**, respectively)	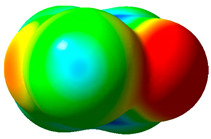	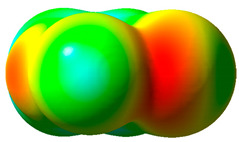
In plane, into the S–S bond	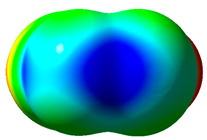	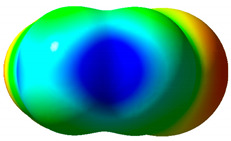

**Table 3:** Experimental and computational bond lengths (Å) of the dithiazolone **2b** *^a^*.

Bond	X-ray Data	Expt Range	B3LYP	BLYP	M06	mPW1PW	PBE	MP2
C5–S1	1.774(2)	1.776–1.772	1.812	1.853	1.810	1.794	1.793	1.793
S1–S2	2.0533(7)	2.0540–2.0526	2.083	2.106	2.068	2.056	2.053	2.050
S2–N3	1.645(2)	1.647–1.643	1.642	1.657	1.630	1.630	1.629	1.635
N3–C4	1.269(3)	1.272–1.266	1.276	1.291	1.271	1.274	1.275	1.297
C5–C4	1.476(2)	1.478–1.474	1.492	1.495	1.486	1.486	1.486	1.480
C5–O	1.208(2)	1.210–1.206	1.197	1.209	1.190	1.194	1.195	1.211
C4–Cl	1.715(2)	1.717–1.713	1.719	1.738	1.710	1.703	1.701	1.702

*^a^* Blue and red bond lengths are within and out of the experimental range, respectively.

**Table 4:** Experimental and computational bond angles (°) of the dithiazolone **2b** *^a^*.

Angles	X-ray Data	Expt Range	B3LYP	BLYP	M06	mPW1PW	PBE1PBE	MP2
C5–S1–S2	93.96(6)	94.02–93.90	93.30	92.81	93.34	93.70	93.75	94.16
S1–S2–N3	98.23(7)	98.16–98.30	97.99	98.41	98.26	98.29	98.35	98.78
S2–N3–C4	115.9(1)	115.8–116.0	117.84	117.45	117.73	117.52	117.42	116.10
N3–C4–C5	123.1(2)	122.9–123.3	121.96	122.76	122.16	121.74	121.75	122.46
N3–C4–Cl	120.5(1)	120.4–120.6	121.13	120.43	121.57	121.34	121.37	120.61
C5–C4–Cl	116.3(1)	116.2–116.4	116.917	116.82	116.27	116.92	116.89	116.93
C4–C5–S1	108.7(1)	108.6–108.8	108.910	108.58	108.51	108.76	108.73	108.51
C4–C5–O	126.6(2)	126.4–126.8	127.493	128.22	127.78	127.45	127.45	127.60
S1–C5–O	124.7(1)	124.6–124.8	123.597	123.20	123.71	123.80	123.82	123.91

*^a^* Blue and red bond angles are within and out of the experimental range, respectively.

**Table 5:** Experimental and computational bond lengths (Å) of the dithiazolethione **2c** *^a^*.

Bonds	X-ray Data	Expt Range	B3LYP	BLYP	M06	mPW1PW	PBE1PBE	MP2
C5–S1	1.740(4)	1.744–1.736	1.749	1.772	1.742	1.735	1.735	1.739
S1–S2	2.066(2)	2.068–2.064	2.095	2.130	2.080	2.066	2.064	2.057
S2–N3	1.642(4)	1.646–1.638	1.637	1.655	1.625	1.625	1.625	1.627
N3–C4	1.283(6)	1.289–1.277	1.284	1.299	1.279	1.281	1.282	1.309
C5–C4	1.470(6)	1.476–1.464	1.471	1.475	1.465	1.466	1.466	1.457
C5–S	1.639(4)	1.643–1.635	1.636	1.651	1.630	1.628	1.629	1.644
C4–Cl	1.718(4)	1.722–1.714	1.720	1.739	1.711	1.703	1.702	1.702

*^a^* Blue and red bond lengths are within and out of the experimental range, respectively.

**Table 6:** Experimental and computational bond angles (°) of dithiazolethione **2c** *^a^*.

Angles	X-ray Data	Expt Range	B3LYP	BLYP	M06	mPW1PW	PBE1PBE	MP2
C5–S1–S2	95.2(1)	95.1–95.3	94.3	94.0	94.4	94.6	94.7	94.9
S1–S2–N3	96.8(2)	96.6–97.0	96.3	96.2	96.6	96.8	96.9	97.6
S2–N3–C4	116.2(3)	115.9–116.5	117.6	117.3	117.5	117.3	117.2	115.8
N3–C4–C5	123.0(4)	122.6–123.4	122.0	122.7	122.1	121.7	121.7	122.3
N3–C4–Cl	118.0(3)	117.7–118.3	119.1	118.5	119.5	119.5	119.5	118.7
C5–C4–Cl	119.0(3)	118.7–119.3	119.0	118.8	118.4	118.8	118.8	119.0
C4–C5–S1	108.7(3)	108.4–109.0	109.7	109.8	109.5	109.6	109.6	109.5
C4–C5–S	126.9(3)	126.6–127.2	128.1	128.8	128.3	127.9	127.8	127.8
S1–C5–S	124.3(2)	124.1–124.5	122.2	121.5	122.2	122.6	122.6	122.7

*^a^* Blue and red bond angles are within and out of the experimental range, respectively.

**Table 7:** NBO bond orders calculated at the B3LYP/def2-TZVPD level of theory for Appel’s salt **1**, dithiazolone **2b** and dithiazolethione **2c**.

Bond	NBO–1	Bond	NBO–2b	Bond	NBO–2c
C5–S1	1.36	C5–S1	1.06	C5–S1	1.15
S1–S2	1.08	S1–S2	1.00	S1–S2	0.98
S2–N3	1.29	S2–N3	1.12	S2–N3	1.13
N3–C4	1.48	N3–C4	1.68	N3–C4	1.64
C5–C4	1.21	C5–C4	1.01	C5–C4	1.07
C5–Cl2	1.22	C5–O	1.77	C5–S	1.66
C4–Cl1	1.14	C4–Cl	1.07	C4–Cl	1.07

**Table 8:** Nucleus-independent chemical shifts (NICS) taken 1 Å over the center of the ring of Appel’s salt **1**, dithiazolone **2b** and dithiazolethione **2c** rings.

System	NICS(1)
Appel’s salt **1**	−9.75
Dithiazolone **2b**	−5.43
Dithiazolethione **2c**	−4.71

## Data Availability

Not applicable.

## Supplementary Materials

The following are available online. Tables S1–S11 and S21–S22: Crystallographic data for **2b**, **2c**–***α*****, 2c**–***β*** and **1**, Figures S1–S3: Crystal packing of **2b**, **2c**–***α*** and **2c**–***β***, Tables S12–S20: Condensed Fukui functions for **2b**, **2c**–***α,* 2c**–***β*** and **1**.
