# Supplementary material for: Crystal Structure and Solid-State Packing of 4-Chloro-5H-1,2,3-dithiazol-5-one and 4-Chloro-5H-1,2,3-dithiazole-5-thione
Source: Molecules. 2021 Sep 28;26(19):5875. doi: 10.3390/molecules26195875 (PMC8512425; doi:10.3390/molecules26195875)
Supplement: Supplementary file 1 [file molecules-26-05875-s001.zip › molecules-1379726-supplementary.pdf]

Crystal Structures and Solid-State Packing of 4-Chloro-5*H*-1,2,3-dithiazol-5-one and 4-Chloro-5*H*-1,2,3-dithiazole-5-thione

Christos P. Constantinides[2,*] Maria Koyioni,[1] Fadwat Bazzi,[2] Maria Manoli,[1] Daniel B. Lawson[2] and Panayiotis A. Koutentis[1]

[1] Department of Chemistry, University of Cyprus, P.O. Box 20537, 1678 Nicosia, Cyprus

[2] Department of Natural Sciences, University of Michigan – Dearborn, 4901 Evergreen Road, Dearborn, MI 48128-1491, United States

* Correspondence e-mail: cconst@umich.edu Fax: +01 3135934937 Tel: +01 3135836728

Supplementary Information

Title	Page no.
**Table S1.** Crystallographic data for 1,2,3-dithiazol-5-ones/thiones **2b**, **2c-*α*** and **2c-*β***.	4
**Table S2.** Intramolecular geometrical parameters for literature 5*H*-1,2,3-dithiazoles **3**, **4** and **5**.	5
**Table S3.** Symmetry operators in crystal packing of dithiazolone **2b** and thiones **2c-*α*** and **2c-*β***.	6
**Figure S1.** Crystal packing of dithiazolone **2b** along the *c*-axis (a) and along the *bc*-diagonal.	7
**Figure S2.** Crystal packing of dithiazolethione **2c-*α*** along the *a*-axis (a); along the *b*-axis (b); along the *ac*-diagonal (c).	8
**Figure S3.** Crystal packing of dithiazolethione **2c-*β*** showing the formation of 2D sheets (a) and the parallel stacking of the sheets along the *a*-axis (b).	9

**Table S4.** Percentage difference between the experimental and computational bond lengths (Å) of the dithiazolone **2b**.	10
**Table S5.** Weighted standard deviation (WSD) between the experimental and computational bond lengths (Å) of the dithiazolone **2b**.	10
**Table S6.** Percentage difference between the experimental and computational bond angles (°) of the dithiazolone **2b**.	11
**Table S7.** Weighted standard deviation (WSD) between the experimental and computational bond angles of the dithiazolone **2b**.	11
**Table S8.** Percentage difference between the experimental and computational bond lengths (Å) of dithiazolethione **2c**.	12
**Table S9.** Weighted standard deviation (WSD) between the experimental and computational bond lengths (Å) of the dithiazolethione **2c**.	12
**Table S10.** Percentage difference between the experimental and computational bond angles (°) of dithiazolethione **2c**.	13
**Table S11.** Weighted standard deviation (WSD) between the experimental and computational bond angles of the dithiazolethione **2c**.	13
**Table S12.** Condensed electrophilic Fukui functions (f^{+}) for dithiazolone **2b**.	14
**Table S13.** Condensed nucleophilic Fukui functions (f^{-}) for dithiazolone **2b**.	14
**Table S14.** Condensed radicalary attack Fukui functions (f^{0}) for dithiazolone **2b**.	15
**Table S15.** Condensed electrophilic Fukui functions (f^{+}) for dithiazolethione **2c**.	15
**Table S16.** Condensed nucleophilic Fukui functions (f^{-}) for dithiazolethione **2c**.	16
**Table S17.** Condensed radicalary attack Fukui functions (f^{0}) for dithiazolethione **2c**.	16
**Table S18.** Condensed electrophilic Fukui functions (f^{+}) of Appel's salt **1**.	17
**Table S19.** Condensed nucleophilic Fukui functions (f^{-}) of Appel's salt **1**.	18

Table S20. Condensed radicalary attack Fukui functions (f^0) of Appel's salt **1**. 18

Table S21. Experimental and computational bond lengths (Å) of the Appel's salt **1**. 19

Table S22. Experimental and computational bond angles (°) of the Appel's salt **1**. 19

Table S1. Crystallographic data for 1,2,3-dithiazol-5-ones/thiones **2b**, **2c-*α*** and **2c-*β***.

	**2b**	**2c-*α***	**2c-*β***
CCDC Number	2106260	2106261	2106262
		**Crystal Data**	
Formula	C_2ClNS_2O	C_2ClNS_3	C_2ClNS_3
Formula weight, g·mol^{-1}	153.60	169.66	169.66
Crystal system	Orthorhombic	Orthorhombic	Triclinic
Space group	*P b c a*	*P b c a*	*P -1*
a, b, c, Å	5.6128(3), 12.4796(9), 14.3419(9)	11.0262(5), 7.2698(3), 13.3111(8)	7.113(5), 13.323(5), 18.343(5)
α, β, γ °	90.00, 90.00, 90.00	90.00, 90.00, 90.00	76.886(5), 82.914(5), 76.429(5)
V, Å^3	1004.59(11)	1067.00(9)	1641.2(14)
Z	8	8	12
ρ_{calc}, g·cm^{-3}	2.031	2.112	2.060
μ, mm^{-1}	13.408 (Cu Ka)	16.122 (Cu Ka)	1.694 (Mo Ka)
F(000)	608	672	1008
Crystal size, mm^3	0.12 × 0.07 × 0.05	0.17 × 0.17 × 0.02	0.11 × 0.03 × 0.01
		**Data Collection** ^{a}	
T, K	100(2)	100(2)	100(2)
λ^a, Å	1.54184	1.54184	0.71073
θ (min, max), °	6.17, 72.06	6.65, 66.96	2.95, 25.00
Dataset (-h, h; -k, k; -l, l)	-5, 6; -14, 15; -13, 17	-13, 12; -3, 8; -15, 15	-5, 8; -15, 15; -21, 21
Meas./ indep. refl. (R_{int})	3093 / 982 (0.0246)	5321 / 953 (0.1001)	10938 / 5782 (0.0911)
Obs. refl. [I > 2σ(I)]	946	944	3131
		**Refinement**	
R_1 ^{b}	0.0261	0.0680	0.0845
wR_2 ^{c}	0.0663	0.1993	0.2120
Goodness of fit on F^2	1.112	1.077	0.981
Min, max resd density, e.Å^{-3}	0.264/ -0.381	0.836/ -1.097	0.228/ -0.806

^{a} Graphite monochromator.

^{b} $R_1 = \Sigma \| F_o | - | F_c \| / \Sigma | F_o |$

^{c} $wR_2 = [\Sigma[w(F_o^2 - F_c^2)^2]/\Sigma[wF_o^2)^2]]^{1/2}$, $w = 1/[\sigma^2(F_o^2) + (m\cdot p)^2 + n\cdot p]$, $p = [\max(F_o^2,0) + 2F_c^2]/3$

Table S2. Intramolecular geometrical parameters for literature 5*H*-1,2,3-dithiazoles **3**, **4** and **5**.

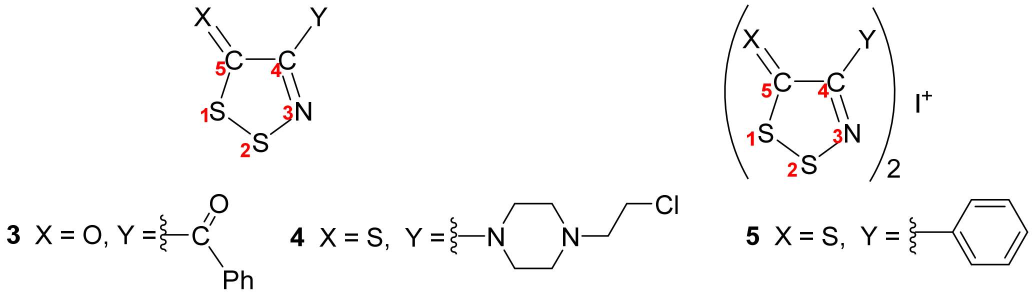

Compound	Bonds	Bond Length (Å)	Angles	Bond Angles (°)
**3**	C5–S1	1.802(2)	C5–S1–S2	93.70(6)
CCDC: 1522822				
	S1–S2	2.0431(6)	S1–S2–N3	98.59(6)
	S2–N3	1.628(1)	S2–N3–C4	118.2(1)
	N3–C4	1.299(2)	N3–C4–C5	119.8(1)
	C5–C4	1.485(2)	N3–C4–Y	119.7(1)
	C5–X	1.211(2)	C5–C4–Y	120.4(1)
	C4–Y	1.502(2)	C4–C5–S1	109.7(1)
			C4–C5–X	128.7(1)
			S1–C5–X	121.6(1)
**4**	C5–S1	1.727(2)	C5–S1–S2	95.14(7)
CCDC: 1426477	S1–S2	2.0619(7)	S1–S2–N3	96.31(6)
	S2–N3	1.644(2)	S2–N3–C4	118.2(1)
	N3–C4	1.309(2)	N3–C4–C5	119.0(2)
	C5–C4	1.473(3)	N3–C4–Y	120.1(2)
	C5–X	1.657(2)	C5–C4–Y	120.7(2)
	C4–Y	1.391(3)	C4–C5–S1	111.2(1)
			C4–C5–X	128.0(1)
			S1–C5–X	120.7(1)
**5** [a]	C5–S1	1.706(6)-1.708(6)	C5–S1–S2	93.3(2)-93.4(2)
CCDC: 2008357	S1–S2	2.066(2)-2.069(2)	S1–S2–N3	97.2(2)
	S2–N3	1.617(6)-1.626(5)	S2–N3–C4	117.7(4)-117.9(4)
	N3–C4	1.309(8)-1.315(8)	N3–C4–C5	118.0(5)-118.2(5)
	C5–C4	1.450(8)-1.454(8)	N3–C4–Y	118.9(5)-119.3(5)
	C5–X	1.693(6)-1.694(6)	C5–C4–Y	122.6(5)-122.9(5)
	C4–Y	1.476(8)-1.483(8)	C4–C5–S1	113.3(4)-113.8(4)
			C4–C5–X	122.6(4)-122.7(4)
			S1–C5–X	123.7(3)-124.0(4)

[a] Bond lengths and angles given for both dithiazole rings in the S–I^+–S complex.

Table S3. Symmetry operators in crystal packing of dithiazolone **2b** and thiones **2c-*α*** and **2c-*β***.

Compound	Symmetry Operators	Description
**2b**	x,y,z	Identity
	1/2-x,-y,1/2+z	2-fold screw axis with direction [0, 0, 1] at 1/4, 0, z with screw component [0, 0, 1/2]
	1/2+x,1/2-y,-z	2-fold screw axis with direction [1, 0, 0] at x, 1/4, 0 with screw component [1/2, 0, 0]
	-x,1/2+y,1/2-z	2-fold screw axis with direction [0, 1, 0] at 0, y, 1/4 with screw component [0, 1/2, 0]
	-x,-y,-z	Inversion at [0, 0, 0]
	1/2+x,y,1/2-z	Glide plane perpendicular to [0, 0, 1] with glide component [1/2, 0, 0]
	1/2-x,1/2+y,z	Glide plane perpendicular to [1, 0, 0] with glide component [0, 1/2, 0]
	x,1/2-y,1/2+z	Glide plane perpendicular to [0, 1, 0] with glide component [0, 0, 1/2]
**2c-*α***	x,y,z	Identity
	1/2-x,-y,1/2+z	2-fold screw axis with direction [0, 0, 1] at 1/4, 0, z with screw component [0, 0, 1/2]
	-x,1/2+y,1/2-z	2-fold screw axis with direction [0, 1, 0] at 0, y, 1/4 with screw component [0, 1/2, 0]
	1/2+x,1/2-y,-z	2-fold screw axis with direction [1, 0, 0] at x, 1/4, 0 with screw component [1/2, 0, 0]
	-x,-y,-z	Inversion at [0, 0, 0]
	1/2+x,y,1/2-z	Glide plane perpendicular to [0, 0, 1] with glide component [1/2, 0, 0]
	x,1/2-y,1/2+z	Glide plane perpendicular to [0, 1, 0] with glide component [0, 0, 1/2]
	1/2-x,1/2+y,z	Glide plane perpendicular to [1, 0, 0] with glide component [0, 1/2, 0]
**2c-*β***	x,y,z	Identity
	-x,-y,-z	Inversion at [0, 0, 0]

Figure S1. Crystal packing of dithiazolone **2b** along the *c*-axis (a) and along the *bc*-diagonal showing the shortest intermolecular contacts (Å).

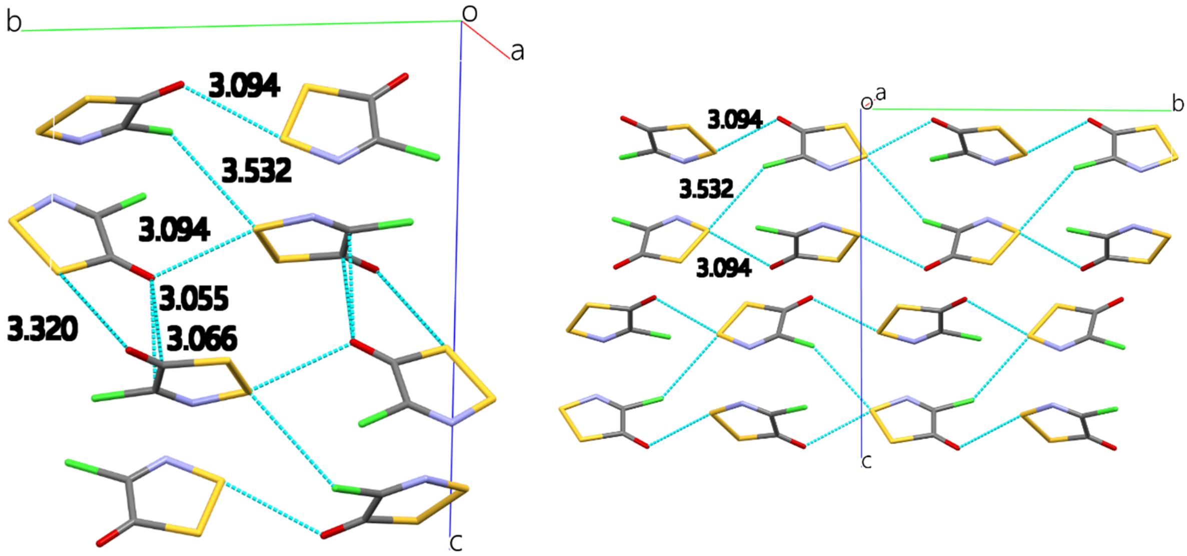

(a) (b)

Figure S2. Crystal packing of dithiazolethione **2c-*α*** along the *a*-axis (a); along the *b*-axis (b); along the *ac*-diagonal (c) showing the shortest intermolecular contacts (Å).

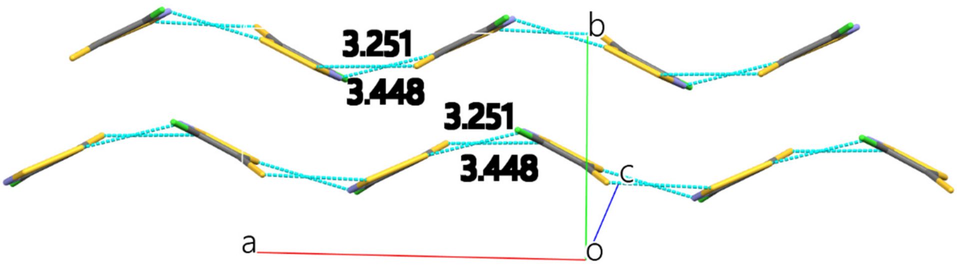

(a)

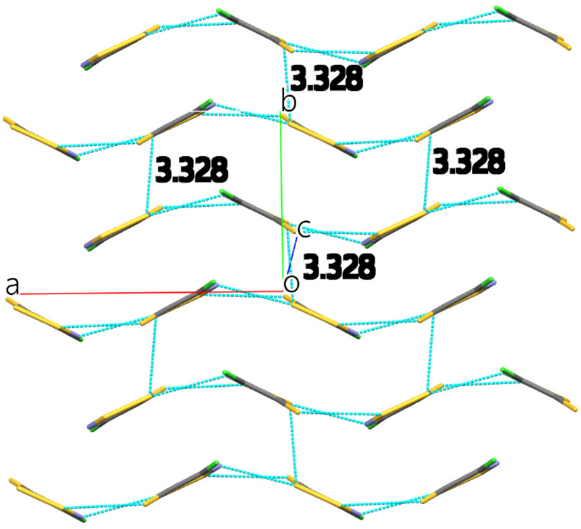

(b)

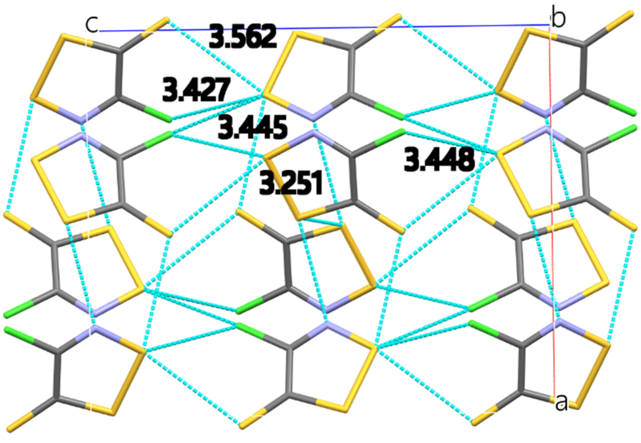

(c)

Figure S3. Crystal packing of dithiazolethione **2c-*β*** showing the formation of 2D sheets (a) and the parallel stacking of the sheets along the *a*-axis (b). Shortest intermolecular contacts (Å) are omitted for clarity purposes. Please see text for comments of intermolecular contacts.

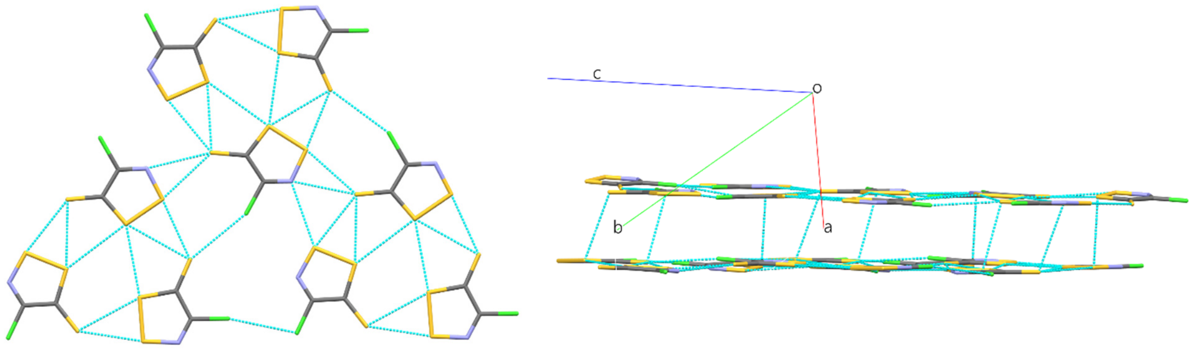

(a) (b)

Table S4. Percentage difference between the experimental and computational bond lengths (Å) of the dithiazolone **2b**.

Bonds	B3LYP	BLYP	M06	mPW1PW	PBE	MP2	average
C5–S1	2.1%	-4.5%	-2.0%	-1.1%	-1.1%	-1.1%	-1.3%
S1–S2	1.4%	-2.6%	-0.7%	-0.1%	0.0%	0.2%	-0.3%
S2–N3	-0.2%	-0.7%	0.9%	0.9%	1.0%	0.6%	0.4%
N3–C4	0.6%	-1.7%	-0.2%	-0.4%	-0.5%	-2.2%	-0.7%
C5–C4	1.1%	-1.3%	-0.7%	-0.7%	-0.7%	-0.3%	-0.4%
C5–O	-0.9%	-0.1%	1.5%	1.2%	1.1%	-0.2%	0.4%
C4–Cl	0.2%	-1.3%	0.3%	0.7%	0.8%	0.8%	0.2%
average	0.6%	-1.7%	-0.1%	0.1%	0.1%	-0.3%	

Table S5. Weighted standard deviation (WSD) between the experimental and computational bond lengths (Å) of the dithiazolone **2b**.

Bonds	B3LYP	BLYP	M06	mPW1PW	PBE	MP2
C5–S1	19.0	39.5	18.0	10.0	9.5	9.5
S1–S2	42.4	75.3	21.0	3.9	-0.4	-4.7
S2–N3	-1.5	6.0	-7.5	-7.5	-8.0	-5.0
N3–C4	2.3	7.3	0.7	1.7	2.0	9.3
C5–C4	8.0	9.5	5.0	5.0	5.0	2.0
C5–O	-5.5	0.5	-9.0	-7.0	-6.5	1.5
C4–Cl	2.0	11.5	-2.5	-6.0	-7.0	-6.5

The values in the table are computed as:

$$(R_{eq,calc} - R_{eq,exptl})/_{uncertainy} = multiple\ of\ the\ \mathrm{WSD}$$

Table S6. Percentage difference between the experimental and computational bond angles (°) of the dithiazolone **2b**.

Angles	B3LYP	BLYP	M06	mPW1PW	PBE	MP2	average
C5–S1–S2	0.7%	1.2%	0.7%	0.3%	0.2%	-0.2%	0.5%
S1–S2–N3	0.2%	-0.2%	0.0%	-0.1%	-0.1%	-0.6%	-0.1%
S2–N3–C4	-1.6%	-1.3%	-1.6%	-1.4%	-1.3%	-0.2%	-1.2%
N3–C4–C5	0.9%	0.3%	0.8%	1.1%	1.1%	0.5%	0.8%
N3–C4–Cl	-0.5%	0.1%	-0.9%	-0.7%	-0.7%	-0.1%	-0.5%
C5–C4–Cl	-0.5%	-0.4%	0.0%	-0.5%	-0.5%	-0.5%	-0.4%
C4–C5–S1	-0.2%	0.1%	0.2%	-0.1%	0.0%	0.2%	0.0%
C4–C5–O	-0.7%	-1.3%	-0.9%	-0.7%	-0.7%	-0.8%	-0.8%
S1–C5–O	0.9%	1.2%	0.8%	0.7%	0.7%	0.6%	0.8%
average	-0.1%	0.0%	-0.1%	-0.1%	-0.1%	-0.1%	

Table S7. Weighted standard deviation (WSD) between the experimental and computational bond angles of the dithiazolone **2b**.

Angles	B3LYP	BLYP	M06	mPW1PW	PBE1PBE	MP2
C5–S1–S2	-11.0	-19.2	-10.3	-4.3	-3.5	3.3
S1–S2–N3	-3.4	2.6	0.4	0.9	1.7	7.9
S2–N3–C4	19.4	15.5	18.3	16.2	15.2	2.0
N3–C4–C5	-5.7	-1.7	-4.7	-6.8	-6.7	-3.2
N3–C4–Cl	6.3	-0.7	10.7	8.4	8.7	1.1
C5–C4–Cl	6.2	5.2	-0.3	6.2	5.9	6.3
C4–C5–S1	2.1	-1.2	-1.9	0.6	0.3	-1.9
C4–C5–O	4.5	8.1	5.9	4.3	4.3	5.0
S1–C5–O	-11.0	-15.0	-9.9	-9.0	-8.8	-7.9

The values in the table are computed as:

$$\left(\angle_{eq,calc} - \angle_{eq,exptl}\right) \big/ uncertainty = multiple\ of\ the\ \mathrm{WSD}$$

Table S8. Percentage difference between the experimental and computational bond lengths (Å) of dithiazolethione **2c**.

Bonds	B3LYP	BLYP	M06	mPW1PW	PBE1PBE	MP2	average
C5–S1	-0.5%	-1.8%	-0.1%	0.3%	0.3%	0.0%	-0.3%
S1–S2	-1.4%	-3.1%	-0.7%	0.0%	0.1%	0.4%	-0.8%
S2–N3	0.3%	-0.8%	1.0%	1.0%	1.1%	0.9%	0.6%
N3–C4	-0.1%	-1.2%	0.3%	0.1%	0.0%	-2.0%	-0.5%
C5–C4	-0.1%	-0.3%	0.3%	0.3%	0.3%	0.9%	0.2%
C5–S	0.2%	-0.7%	0.5%	0.6%	0.6%	-0.3%	0.2%
C4–Cl	-0.1%	-1.2%	0.4%	0.9%	1.0%	0.9%	0.3%
average	-0.2%	-1.3%	0.3%	0.5%	0.5%	0.1%	

Table S9. Weighted standard deviation (WSD) between the experimental and computational bond lengths (Å) of the dithiazolethione **2c**.

Bonds	B3LYP	BLYP	M06	mPW1PW	PBE	MP2
C5–S1	2.3	8.0	0.6	-1.1	-1.3	-0.1
S1–S2	14.5	32.0	7.1	0.1	-1.2	-4.3
S2–N3	-1.2	3.3	-4.1	-4.3	-4.4	-3.7
N3–C4	0.2	2.7	-0.7	-0.3	-0.1	4.3
C5–C4	0.2	0.8	-0.8	-0.6	-0.7	-2.2
C5–S	-0.8	3.0	-2.2	-2.7	-2.5	1.2
C4–Cl	0.5	5.3	-1.8	-3.7	-4.1	-4.0

Table S10. Percentage difference between the experimental and computational bond angles (°) of dithiazolethione **2c**.

Angles	B3LYP	BLYP	M06	mPW1PW	PBE1PBE	MP2	average
C5–S1–S2	0.9%	1.3%	0.9%	0.6%	0.6%	0.4%	0.8%
S1–S2–N3	0.5%	0.6%	0.2%	0.0%	-0.1%	-0.8%	0.1%
S2–N3–C4	-1.2%	-0.9%	-1.1%	-0.9%	-0.8%	0.4%	-0.8%
N3–C4–C5	0.8%	0.2%	0.8%	1.0%	1.0%	0.6%	0.7%
N3–C4–Cl	-0.9%	-0.4%	-1.3%	-1.3%	-1.3%	-0.6%	-1.0%
C5–C4–Cl	0.0%	0.2%	0.5%	0.2%	0.2%	0.0%	0.2%
C4–C5–S1	-0.9%	-1.0%	-0.7%	-0.8%	-0.8%	-0.7%	-0.8%
C4–C5–S	-0.9%	-1.5%	-1.1%	-0.8%	-0.7%	-0.7%	-1.0%
S1–C5–S	1.7%	2.3%	1.7%	1.4%	1.4%	1.3%	1.6%
average	0.0%	0.1%	0.0%	-0.1%	-0.1%	0.0%	

Table S11. Weighted standard deviation (WSD) between the experimental and computational bond angles of the dithiazolethione **2c**.

Angles	B3LYP	BLYP	M06	mPW1PW	PBE	MP2
C5–S1–S2	-8.6	-12.0	-8.2	-5.8	-5.3	-3.4
S1–S2–N3	-2.3	-2.8	-1.2	0.0	0.4	4.1
S2–N3–C4	4.8	3.6	4.3	3.5	3.2	-1.5
N3–C4–C5	-2.6	-0.7	-2.3	-3.2	-3.2	-1.7
N3–C4–Cl	3.6	1.7	5.1	5.0	5.1	2.3
C5–C4–Cl	-0.1	-0.7	-2.0	-0.8	-0.8	0.0
C4–C5–S1	3.4	3.5	2.7	2.9	2.9	2.5
C4–C5–S	4.0	6.2	4.6	3.2	3.2	3.1
S1–C5–S	-10.6	-14.2	-10.5	-8.7	-8.6	-7.9

Table S12. Condensed electrophilic Fukui functions (f^+) (initial site of nucleophilic attack) with the B3LYP, BLYP, M06, mPW1PW, and PBE1PBE and the def2-TZVPD basis set for dithiazolone **2b**.[a]

Atom	B3LYP	BLYP	M06	mPW1PW	PBE
S1	0.217	0.223	0.217	0.210	0.209
S2	0.343	0.305	0.348	0.353	0.352
N3	0.003	0.024	-0.004	-0.002	-0.002
C4	0.089	0.075	0.097	0.095	0.095
C5	-0.014	0.000	-0.019	-0.018	-0.018
Cl	0.186	0.206	0.186	0.181	0.181
O	0.175	0.167	0.174	0.181	0.182

[a] Blue and red values indicate sites of highest and lowest electrophilicity, respectively.

Table S13. Condensed nucleophilic Fukui functions (f^-) (initial site of electrophilic attack) with the B3LYP, BLYP, M06, mPW1PW, and PBE1PBE and the def2-TZVPD basis set for dithiazolone **2b**.[a]

Atom	B3LYP	BLYP	M06	mPW1PW	PBE1PBE
S1	0.182	0.192	0.182	0.181	0.181
S2	0.182	0.193	0.185	0.182	0.183
N3	0.171	0.160	0.172	0.172	0.172
C4	0.110	0.102	0.109	0.113	0.113
C5	0.089	0.084	0.088	0.086	0.085
Cl	0.117	0.121	0.120	0.117	0.116
O	0.149	0.147	0.145	0.149	0.149

[a] Blue and red values indicate sites of highest and lowest nucleophilicity, respectively.

Table S14. Condensed radicalary attack Fukui functions (f^0) (initial site of radical attack) with the B3LYP, BLYP, M06, mPW1PW, and PBE1PBE and the def2-TZVPD basis set for dithiazolone **2b**.[a]

Atom	B3LYP	BLYP	M06	mPW1PW	PBE1PBE
S1	0.200	0.208	0.200	0.196	0.195
S2	0.262	0.249	0.266	0.268	0.268
N3	0.087	0.092	0.084	0.085	0.085
C4	0.100	0.089	0.103	0.104	0.104
C5	0.038	0.042	0.034	0.034	0.034
Cl	0.151	0.164	0.153	0.149	0.149
O	0.162	0.157	0.160	0.165	0.165

[a] Blue and red values indicate sites of highest and lowest activity for radical attack, respectively.

Table S15. Condensed electrophilic Fukui functions (f^+) (initial site of nucleophilic attack) with the B3LYP, BLYP, M06, mPW1PW, and PBE1PBE and the def2-TZVPD basis set for dithiazolethione **2c**.[a]

Atom	B3LYP	BLYP	M06	mMP1MP	PBE1PBE
S1	0.201	0.181	0.202	0.200	0.199
S2	0.273	0.241	0.273	0.276	0.275
N3	0.024	0.039	0.019	0.021	0.021
C4	0.052	0.034	0.057	0.056	0.056
C5	-0.056	-0.058	-0.070	-0.063	-0.063
Cl	0.117	0.131	0.118	0.113	0.113
S	0.389	0.432	0.402	0.398	0.399

[a] Blue and red values indicate sites of highest and lowest electrophilicity, respectively.

Table S16. Condensed nucleophilic Fukui functions (f^-) (initial site of electrophilic attack) with the B3LYP, BLYP, M06, mPW1PW, and PBE1PBE and the def2-TZVPD basis set for dithiazolethione **2c**.[a]

Atom	B3LYP	BLYP	M06	mMP1MP	PBE1PBE
S1	0.171	0.174	0.171	0.172	0.172
S2	0.173	0.181	0.175	0.173	0.173
N3	0.136	0.131	0.135	0.136	0.137
C4	0.032	0.041	0.026	0.031	0.031
C5	0.074	0.062	0.075	0.075	0.074
Cl	0.089	0.095	0.090	0.088	0.088
S	0.326	0.317	0.330	0.327	0.326

[a] Blue and red values indicate sites of highest and lowest nucleophilicity, respectively.

Table S17. Condensed radicalary attack Fukui functions (f^0) (initial site of radical attack) with the B3LYP, BLYP, M06, mPW1PW, and PBE1PBE and the def2-TZVPD basis set for dithiazolethione **2c**.[a]

Atom	B3LYP	BLYP	M06	mMP1MP	PBE1PBE
S1	0.186	0.178	0.186	0.186	0.186
S2	0.223	0.211	0.224	0.224	0.224
N3	0.080	0.085	0.077	0.078	0.079
C4	0.042	0.037	0.041	0.043	0.044
C5	0.009	0.002	0.002	0.006	0.006
Cl	0.103	0.113	0.104	0.100	0.100
S	0.358	0.374	0.366	0.362	0.362

[a] Blue and red values indicate sites of highest and lowest activity for radical attack, respectively.

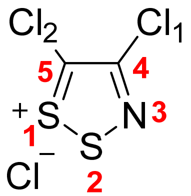

1

Table S18. Condensed electrophilic Fukui functions (f^{+}) (initial site of nucleophilic attack) with the B3LYP, BLYP, M06, mPW1PW, and PBE1PBE and the def2-TZVPD basis set for the Appel's salt **1**.[a]

Atom	B3LYP	BLYP	M06	mPW1PW	PBE
S1	0.231	0.230	0.234	0.233	0.233
S2	0.243	0.238	0.247	0.245	0.245
C4	0.127	0.113	0.126	0.128	0.128
C5	-0.008	0.004	-0.010	-0.009	-0.009
N3	0.134	0.129	0.129	0.134	0.135
Cl2	0.108	0.116	0.109	0.107	0.107
Cl1	0.164	0.170	0.165	0.162	0.161

[a] Blue and red values indicate sites of highest and lowest electrophilicity, respectively.

Table S19. Condensed nucleophilic Fukui functions (f^-) (initial site of electrophilic attack) with the B3LYP, BLYP, M06, mPW1PW, and PBE1PBE and the def2-TZVPD basis set for the Appel's salt **1**.[a]

Atom	B3LYP	BLYP	M06	mPW1PW	PBE1PBE
S1	0.281	0.263	0.291	0.287	0.287
S2	0.110	0.126	0.105	0.103	0.103
C4	0.027	0.020	0.033	0.032	0.032
C5	0.038	0.036	0.045	0.039	0.039
N3	0.019	0.031	0.014	0.016	0.016
Cl2	0.312	0.309	0.301	0.308	0.308
Cl1	0.213	0.216	0.211	0.215	0.216

[a] Blue and red values indicate sites of highest and lowest nucleophilicity, respectively.

Table S20. Condensed radicalary attack Fukui functions (f^0) (initial site of radical attack) with the B3LYP, BLYP, M06, mPW1PW, and PBE1PBE and the def2-TZVPD basis set for the Appel's salt **1**.[a]

Atom	B3LYP	BLYP	M06	mPW1PW	PBE1PBE
S1	0.256	0.246	0.263	0.260	0.260
S2	0.176	0.182	0.176	0.174	0.174
N3	0.077	0.067	0.080	0.080	0.080
C4	0.015	0.020	0.018	0.015	0.015
C5	0.077	0.080	0.072	0.075	0.075
Cl2	0.210	0.213	0.205	0.207	0.207
Cl1	0.188	0.193	0.188	0.188	0.189

[a] Blue and red values indicate sites of highest and lowest activity for radical attack, respectively.

Table S21. Experimental and computational bond lengths (Å) of the Appel's salt **1**.[a]

Bond	X-Ray Data	Expt Range	B3LYP	BLYP	M06	mPW1PW	PBE	MP2
C5–S1	1.674(4)	1.670-1.678	1.691	1.711	1.684	1.681	1.681	1.694
S1–S2	2.032(1)	2.031-2.033	2.069	2.112	2.058	2.040	2.038	2.024
S2–N3	1.611(3)	1.608-1.614	1.592	1.609	1.582	1.583	1.583	1.591
N3–C4	1.301 (5)	1.296-1.306	1.310	1.324	1.304	1.307	1.308	1.335
C5–C4	1.405(5)	1.400-1.410	1.431	1.443	1.426	1.427	1.427	1.418
C5–Cl2	1.681(4)	1.677-1.685	1.675	1.690	1.665	1.663	1.662	1.675
C4–Cl1	1.709(4)	1.705-1.713	1.693	1.708	1.683	1.679	1.678	1.677

[a] Blue and red bond lengths are within and out of the experimental range, respectively.

Table S22. Experimental and computational bond angles (°) of the Appel's salt **1**.[a]

Angles	X-Ray Data	Expt Range	B3LYP	BLYP	M06	mPW1PW	PBE1PBE	MP2
C5–S1–S2	93.0(1)	93.1-92.9	92.07	91.41	91.95	92.42	92.44	92.54
S1–S2–N3	97.4(1)	97.5-97.3	97.44	97.43	97.66	97.91	97.99	99.29
S2–N3–C4	116.3(3)	116.0-116.6	118.25	118.16	118.25	117.89	117.79	115.78
N3–C4–C5	118.7(3)	119.0-118.4	117.09	117.44	117.01	116.97	116.98	117.88
N3–C4–Cl2	119.7(3)	120.0-119.4	121.04	120.72	121.53	121.32	121.35	120.23
C5–C4–Cl2	121.6(3)	121.9-121.3	121.87	121.85	121.46	121.71	121.67	121.90
C4–C5–S1	114.6(3)	114.9-114.3	115.10	115.56	115.14	114.82	114.79	114.52
C4–C5–Cl1	125.7(3)	126.0-125.4	124.37	124.29	124.20	124.38	124.38	124.86
S1–C5–Cl1	119.7(2)	119.9-119.5	120.53	120.15	120.66	120.79	120.83	120.63

[a] Blue and red bond angles are within and out of the experimental range, respectively.
